# PAM identification by CRISPR-Cas effector complexes: diversified mechanisms and structures

**DOI:** 10.1080/15476286.2018.1504546

**Published:** 2018-09-18

**Authors:** Daniel Gleditzsch, Patrick Pausch, Hanna Müller-Esparza, Ahsen Özcan, Xiaohan Guo, Gert Bange, Lennart Randau

**Affiliations:** a Prokaryotic Small RNA Biology Group, Max-Planck-Institute for terrestrial Microbiology & LOEWE Center for synthetic Microbiology (Synmikro), Marburg, Germany; b Philipps-University-Marburg, LOEWE Center for synthetic Microbiology (Synmikro) & Faculty of Chemistry, Marburg, Germany

**Keywords:** CRISPR, Cas proteins, DNA recognition, PAM, ribonucleoproteins

## Abstract

Adaptive immunity of prokaryotes is mediated by CRISPR-Cas systems that employ a large variety of Cas protein effectors to identify and destroy foreign genetic material. The different targeting mechanisms of Cas proteins rely on the proper protection of the host genome sequence while allowing for efficient detection of target sequences, termed protospacers. A short DNA sequence, the protospacer-adjacent motif (PAM), is frequently used to mark proper target sites. Cas proteins have evolved a multitude of PAM-interacting domains, which enables them to cope with viral anti-CRISPR measures that alter the sequence or accessibility of PAM elements. In this review, we summarize known PAM recognition strategies for all CRISPR-Cas types. Available structures of target bound Cas protein effector complexes highlight the diversity of mechanisms and domain architectures that are employed to guarantee target specificity.

## Introduction

Bacteria and Archaea are constantly exposed to foreign genetic material and the invasion of lytic viruses. Consequently, they have evolved numerous defense mechanisms, including CRISPR (clustered regularly interspaced short palindromic repeats)-Cas (CRISPR associated) systems. CRISPR-Cas are adaptive immune systems that utilize short RNA molecules, called CRISPR RNAs (crRNAs), to identify and degrade foreign DNA or RNA [1,2]. The crRNAs contain a variable sequence, termed spacer, that can be derived from previously encountered mobile genetic elements. They form effector complexes with different Cas protein family members to interfere with foreign nucleic acids, e.g. viral genomes. During a recurring infection, these interference complexes will recognize the matching sequence of the viral protospacer and bind to it via base-complementarity with the crRNA. Target binding usually results in the nucleolytic destruction of the viral genetic material. New spacers can be acquired from viral genomes by a process called adaptation, in which the conserved proteins Cas1 and Cas2 insert new spacers in the extending CRISPR locus [3–5].

Besides their natural function of prokaryotic immunity, CRISPR-Cas mechanisms have been adapted to allow for the design of highly efficient genome manipulation tools. Most notable is the CRISPR-Cas9 system that relies on the single effector protein Cas9 in combination with a crRNA-derived single-guide RNA (sgRNA) for target interference. Since its discovery, the CRISPR-Cas9 system has revolutionized genome-editing and transcription regulation approaches [6–12]. CRISPR-Cas systems are highly diverse and novel effector Cas proteins are continuously evaluated for their applicability. Notable examples are the RNA-guided DNase Cas12a [13] and the RNA-guided RNase Cas13a [14].

Target recognition relies on a protospacer adjacent motif (PAM). This short, conserved sequence of 2–5 bp is located next to target DNA and required to discriminate between ‘self’ and ‘non-self’. PAM motifs are not present near spacers of the CRISPR locus to avoid autoimmunity and cleavage of the host genome [15].

PAM elements were discovered by computational analyses as conserved sequences near protospacers that match spacers within CRISPR loci [16]. Later, these motifs were also shown to be recognized during the interference step [17,18]. PAM elements are used to locate bona fide targets in a model first described in *E. coli* [18]. Cas protein surveillance complexes can efficiently scan long DNA sequences, e.g. viral genomes, for the presence of PAM sequences. Specific Cas proteins recognize and bind the PAM sequence and unwind the adjacent dsDNA helix. The opened DNA becomes available for hybridization with the crRNA, producing a triple-stranded R-loop structure. Seed sequences near these PAM elements are interrogated for complementarity with the crRNA spacer to induce full base pairing and subsequent interference [19–24]. It should be noted that some CRISPR-Cas systems target RNA instead of dsDNA and consequently do not require a PAM to specify the site of dsDNA unwinding [25].

PAM sequences are initially recognized during the acquisition of new spacers. Here, the conserved proteins of the acquisition machinery, Cas1 and Cas2, sometimes in combination with the interference complex, recognize the PAM sequence and ensure that the newly incorporated spacer is able to target the invading DNA [4,26]. In some CRISPR-Cas systems, PAM-dependent spacer precursor trimming by the nuclease Cas4 is required for correct spacer uptake [27–30].

As adaptation and interference stages employ different molecular mechanisms, the stringency of PAM sequence recognition is not necessarily identical for both processes. Therefore, PAM elements have been proposed to be divided into spacer acquisition motifs (SAMs) and target interference motifs (TIMs) [31]. In this definition, a SAM element is the functional motif associated with the protospacer that is recognized by spacer acquisition machinery, prior to protospacer excision. The TIM element is the functional motif associated with the protospacer that is recognized by the interference complex. Multiple possible TIM sequences were shown to exist for one PAM and strand-specificity is frequently observed [6,32].

The coevolution of antiviral CRISPR-Cas systems and viral anti-CRISPR measures has resulted in many different types and mechanisms of CRISPR-Cas systems. An enormous variety of Cas protein families is observed. To this date, two classes of CRISPR-Cas systems with multiple types and subtypes have been classified [33]. A multisubunit protein complex (Class 1) or a single protein (Class 2) defines the two classes as effector units. The two classes are further separated into six main types with different signature Cas proteins responsible for target cleavage. Multiple subtypes exist that have evolved different ways of crRNA processing and effector complex formation. Likewise, the different types have evolved various strategies of recognizing PAM elements in foreign genetic material. Alternative PAM readout mechanisms are proposed to ensure evasion of viral countermeasures. One striking example is the discovery of viral anti-CRISPR proteins that have been shown to inhibit interference of specific effector complexes [34–40]. The different readout mechanisms in various CRISPR-Cas types also allow for differences in PAM recognition stringency. The ability to recognize multiple PAM sequences renders the immune system more effective against mutations of the PAM sequence, which would otherwise represent a straightforward viral escape strategy [41–44].

## Identification of PAM sequences

To identify all possible PAMs for various CRISPR-Cas systems, different screening methods have been designed. These PAM prediction methods are either based on *in silico, in vivo* or *in vitro* approaches.

The first approaches to identify PAM sequences relied on alignments of protospacers to identify consensus PAM elements [16,45]. Web tools were created to extract spacer sequences (e.g. CRISPRFinder) [46] and to identify potential target sequences (e.g. CRISPRTarget) [47]. This *in silico* approach represents a fast and easy way to identify potential PAMs but relies on the availability of sequenced phages genomes, which is often missing for non-model organisms. It also does not allow distinction between SAMs and TIMs or recognizes mutations that may be present in the PAM.

An experimental approach for PAM identification involves plasmid depletion assays. Here, a randomized DNA stretch is inserted adjacent to a target sequence within a plasmid that is transformed into a host with an active CRISPR-Cas system. Plasmids are retained if the ‘inactive’ PAM is not recognized, allowing for their recognition via next-generation sequencing [48–50]. This approach requires extensive library coverage to identify the depleted sequences, representing functional PAM elements. Alternatively, PAM sequences can be screened by a recently developed high-throughput *in vivo* method called PAM-SCANR (PAM screen achieved by NOT-gate repression). In this approach, a catalytically dead Cas9 variant (dCas9) is added to a target library. If binding to a functional PAM occurs, expression of *gfp* is diminished. Subsequent fluorescence activated cell sorting (FACS), plasmid purification and sequencing identifies all functional PAM motifs [51]. *In vitro* approaches are based on cleavage of target DNA libraries with multiple PAM sequences and their consecutive sequencing. Positive screening can be performed by sequencing enriched cleavage products [52,53] while negative screening can be achieved by sequencing all remaining uncleaved targets [13]. Benefits of these *in vitro* approaches are the input of larger initial libraries and a better control over the reaction conditions. Possible downsides are the requirement of purified, stable effector complexes and the need to maintain *in vivo* activity in the experimental conditions [53].

Different methods exist to present and visualize PAM sequences of promiscuous CRISPR-Cas systems. The most common ways are consensus sequences and sequence logos but suitable underrepresented PAMs might be missed. Tables can be used to summarize all information but do not represent an easily accessible visualization method. Recently, Krona plots were used to depict all individual PAM sequences with enrichment scores. The visualization method was termed PAM Wheel [51].

In recent years, several structures of target bound effector complexes were solved and provided molecular insights into the varying mechanisms of PAM recognition. In addition, factors that contribute to the specificity of PAM recognition domains have been elucidated. In this review, we present an up-to-date overview of (i) the known diversity of PAM recognition structures found in nature and (ii) approaches to modify PAM specificity.

## PAM recognition by adaptation modules

**Figure 1.**
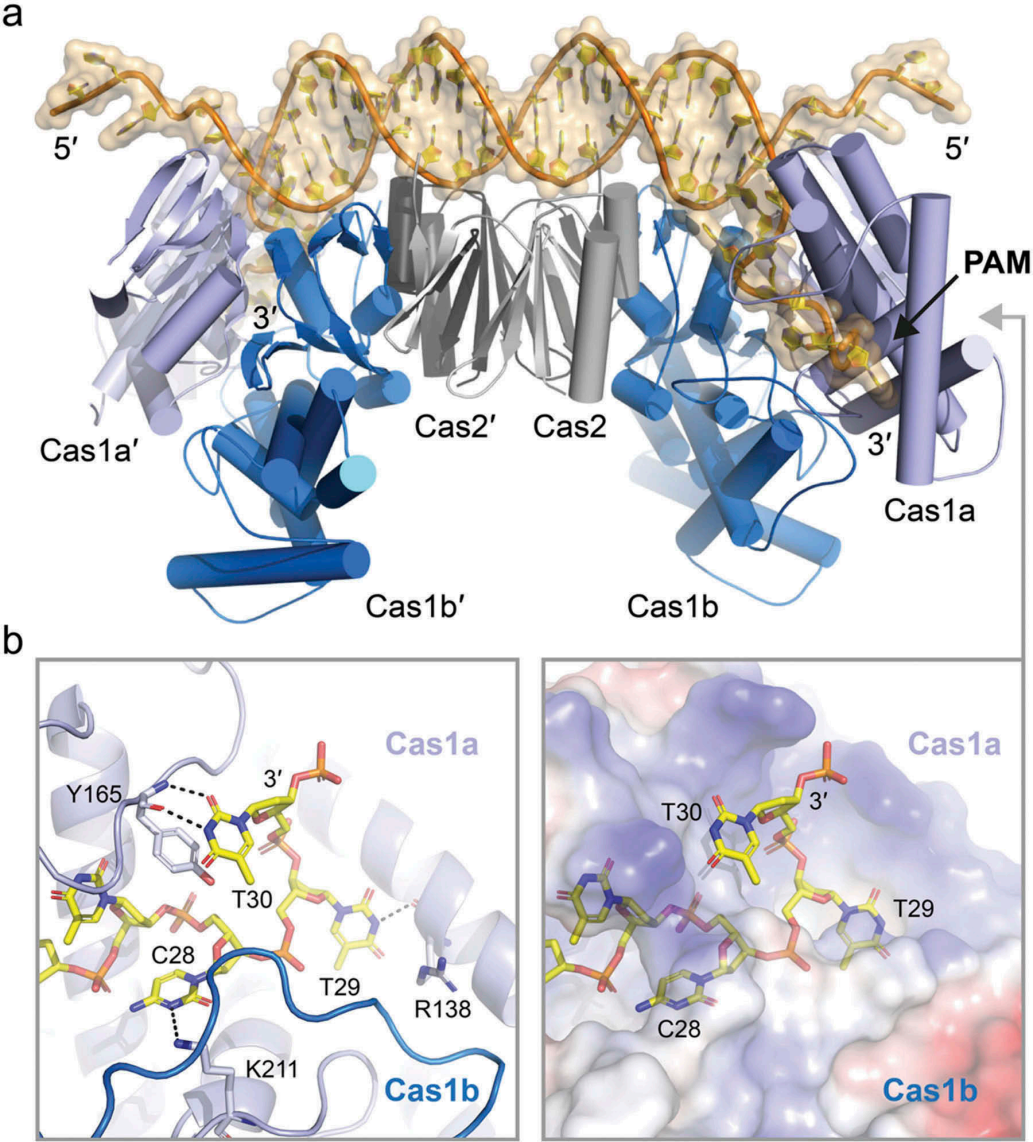
PAM recognition by Cas1-Cas2. a: Crystal structure of the *E. coli* Cas1-Cas2 bound to a dual-forked PAM containing DNA (PDB: 5DQZ [60]). Two copies of Cas2 (light and dark grey) bridge two juxtaposed dimers of Cas1 (light and dark blue). Association of the B-from DNA duplex (orange surface representation) on top of the complex induces bending of the DNA. Cas1a and Cas1b enclose the PAM complementary 3′-overhang (PAM) of the forked DNA. b: Close up on the CTT 3′-overhang (yellow) specific binding pocket formed by Cas1a (light blue) and Cas1b (dark blue). Left: Base specific hydrogen bonding confers specificity (black dotted lines). Right: Surface charge representation of the binding pocket. Cas1a and Cas1b tightly enclose the hook shaped CTT 3′-overhang to provide specificity. Notably, while purine bases would sterically clash with the binding pocket, only pyrimidine bases can be accommodated.

CRISPR-Cas systems employ adaptation modules to process foreign DNA for its integration into expanding CRISPR arrays [41,54–56]. Two modes of adaptation have been observed. The naïve adaptation process integrates sequences that have not been encountered before. In contrast, primed adaptation results in the integration of sequences that partially match pre-existing spacers [41,42,54]. The two conserved Cas1 and Cas2 proteins are essential components of adaptation modules [3,5,57–60] and are sufficient for PAM-dependent protospacer selection and cleavage during naïve adaptation. Cas1 and Cas2 have been shown to form a stable complex consisting of two asymmetric dimers of the metal-dependent DNase Cas1 that are linked by a central Cas2 dimer [3,5,57,59]. A crystal structure of the *Escherichia coli* type I-E Cas1-Cas2 proteins in complex with a dual-forked DNA substrate was obtained [58,60]. The central portion of the DNA substrate duplex binds to the positively charged surface of the Cas2 dimer and the single-stranded DNA overhangs insert into the C-terminal domain of one Cas1 subunit of each Cas1 dimer (Figure 1(a)). Three PAM nucleotides (5′-CTT-3′ in the target strand) are positioned into a base specific pocket provided by the C-terminal domains of the two Cas1 proteins [60]. Efficient protospacer binding relies on the presence of single-stranded overhangs and results in conformational changes as both Cas1 dimers rotate in opposite directions. This movement facilitates cleavage of the target DNA strands generating protospacers with a fixed length (Figure 1(b)).

Recent studies have also revealed that the nuclease Cas4, which is widely conserved among type I, II and V systems [61], assists in PAM-dependent adaptation. Initial DNA processing of spacer precursors (prespacers) by Cas4 ensures the insertion of spacers with a correct size and a defined orientation to prevent the uptake of non-functional spacers [27–29]. In type I-D systems, it has been shown that Cas4 shortens the length of the prespacer and selects molecules with a bias for PAM-compatible sequences [28]. In the type I-C system of *B. halodurans*, Cas4 interacts with Cas1 and prohibits the uptake of unprocessed prespacers [29]. *P. furiosus* contains two different Cas4 proteins that trim prespacers on opposite ends by PAM and downstream motif recognition [30]. While this trimming reaction by Cas4 has been shown to be PAM-dependent, the molecular recognition mechanism remains unknown. CRISPR-Cas systems that do not contain Cas4 proteins might replace this process by RecBCD or Cas3 activities [28,29,62–64].

In type I-F CRISPR-Cas systems, Cas2 is fused to the DNA nuclease Cas3 and adaptation is carried out by a Cas1/Cas2-3 complex [4,64]. The structure of this complex was recently solved by electron microscopy in both *Pectobacterium atrosepticum* and *Pseudomonas aeruginosa* [65,66]. Electron microscopy images of the *P. atrosepticum* Cas1/Cas2-3 complex suggest that the two Cas3 domains form a groove in which the protospacer binds to Cas1/Cas2 [65]. Protospacer integration was observed *in vitro* in the absence of Cas3 activity, which suggests that naïve adaptation relies on other mechanisms to generate protospacer precursors. However, primed adaptation could benefit from the spatial proximity of the active sites of Cas3 and Cas1 in the type I-F adaptation module. It has been proposed that cleavage products of Cas3 might provide protospacer precursors for the Cas1-Cas2 complex [65]. Therefore, PAM recognition plays a critical role in priming which would also be relevant for CRISPR-Cas systems that do not exhibit Cas2-3 fusions. In type I-E systems, Cas3 was proposed to generate DNA fragments, which would be channeled to Cas1/2 to select for PAM matches within this protospacer precursor pool [63,67]. Escape mutations in the PAM sequence have been shown to abolish recruitment of Cas3 to target-bound Cascade unless Cas1 and Cas2 are present. In this PAM-independent pathway, a priming complex helps to select spacers for priming, similar to the above mentioned Cas1/Cas2-3 complex [20]. Recently, direct evidence of this primed acquisition complex (PAC), consisting of Cascade, Cas1/Cas2 and Cas3 was provided by single-molecule imaging in *Thermobifida fusca* [68].

These observations summarize similarities between type I CRISPR-Cas adaptation modules. However, type II-mediated adaptation requires additional components for PAM selection. It was shown that the single effector DNA nuclease Cas9 of the *Streptococcus pyogenes* type II-A CRISPR-Cas system is involved in adaptation. Cas9 recognizes a 5ʹ-NGG-3ʹ PAM sequence during interference. Mutation of its PAM recognition motif resulted in altered PAM specificity and newly acquired spacers corresponded to the altered PAM choice. Based on these results, Cas9 was proposed to provide PAM specificity to the adaptation process in type II CRISPR-Cas systems [69]. The PAM recognition mechanism of Cas9 proteins will be discussed in greater detail below.

## PAM recognition by type i crispr-cas effector complexes

**Figure 2.**
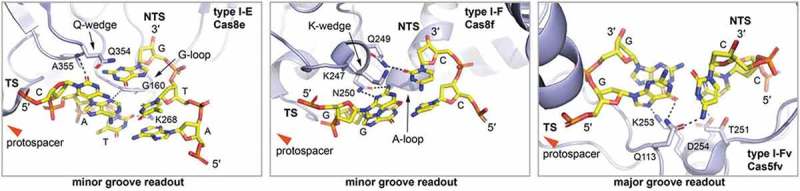
PAM recognition by type I Cascades. Left: Close up on the PAM interacting region of the *E. coli* type I-E Cascade subunit Cas8e (light blue) (PDB: 5H9E [75]). Cas8e promiscuously recognizes the ATG PAM (yellow) via a set of polar interactions (black dashed lines) from the DNA minor groove. The glycine rich loop (G-loop) recognizes the second base pair of the PAM. The Q-wedge might assist in target strand (TS) protospacer displacement from the non-target strand (NTS) protospacer complementary sequence. The red arrow indicates the direction of the protospacer. Middle: Close up on the PAM interacting region of the *P. aeruginosa* type I-F Cascade subunit Cas8f (light blue) (PDB: 6B44 [38]). Cas8e specifically recognizes the GG PAM (yellow) via a set of polar interactions (black dashed lines) from the DNA minor groove. The alanine rich loop (A-loop) recognizes the second base pair of the PAM. The K-wedge might assist in target strand (TS) protospacer displacement from the non-target strand (NTS) protospacer complementary sequence. The red arrow indicates the direction of the protospacer. Right: Close up on the PAM interacting region of the *S. putrefaciens* type I-Fv Cascade subunit Cas5fv (light blue) (PDB: 5O6U [82]). Cas5fv specifically recognizes the GG PAM (yellow) via a set of polar interactions (black dashed lines) from the DNA major groove. Base pairing of the first PAM GC base pair is distorted by Q113. The red arrow indicates the direction of the protospacer.

Type I CRISPR-Cas systems utilize an interference complex called Cascade (CRISPR associated complex for antiviral defense) for target identification [70,71]. These systems are widespread in nature with eight subtypes described so far [33]. The best characterized of these subtypes is the type I-E system from *E. coli* and several crystal structures of the Cascade complex are available [72–75]. This Cascade complex consists of a 61 nt mature crRNA and five Cas proteins with an uneven stoichiometry. A large subunit (Cse1 or Cas8e) and a dimer of small subunits (Cse2) have been shown to mediate PAM recognition and DNA strand guidance to achieve R-loop formation. The molecular mechanism of these processes has been observed in a crystal structure of Cascade bound to an R-loop mimic [75]. The 5′-ATG-3′ PAM is recognized as a double-stranded DNA stretch from its minor groove side by three distinct structural features that are present in the N-terminal domain of the large subunit Cas8e: a glutamine wedge, a glycine loop and a lysine finger (Figure 2). After sampling for a PAM element, the glutamine wedge is inserted towards the first two nucleotides of the protospacer and sterically disrupts them. The dsDNA then melts in a bidirectional fashion and DNA:crRNA heteroduplex formation is facilitated. The C-terminal domain of Cas8e and the Cse2 dimer exhibit conformational rearrangements upon full R-loop formation which leads to recruitment of the Cas3 nuclease for interference.

The steric displacement of the first two protospacer nucleotides in the target strand by the glutamine wedge forces them to rotate outwards. The −2 position of the PAM is promiscuous as a shape readout mechanism rejects only G bases on the target strand [76]. Here, the glycine loop introduces DNA bending. The −3 position has a strong preference for pyrimidines on the target strand due to favorable electrostatic interactions with the lysine finger.

The structure of target-bound type I-E Cascade from *Thermobifida fusca* was solved via cryo-EM [77] and revealed differences in PAM readout, even though the preferred PAM (5ʹ-AAG-3 ʹ) is identical to *E. coli* Cascade. The *T. fusca* Cascade was shown to preferentially form contacts with the non-target strand. The glycine loop is longer and a three amino acid SGM motif is used to read all three PAM base pairs. PAM recognition at the −1 position occurs at the non-target strand in *T. fusca* Cascade and is less stringent compared to *E.coli* Cascade, which contacts both strands.

PAM recognition in type I-E was observed to be more promiscuous in comparison to other type I or type II systems. Five different PAM sequences have been shown to lead to clear CRISPR interference and additional 21 PAM sequences were found to allow acquisition of new spacers [76].

Detailed information about PAM readout mechanisms is also available for Cascade assemblies that belong to subtype I-F. The type I-F *Pseudomonas aeruginosa* Cascade targets foreign DNA with a PAM element consisting of two consecutive G-C base pairs [78]. In contrast to type I-E Cascade, this complex is missing the small subunits and consists of only four Cas proteins [79]. The structure of this Cascade assembly was recently solved by cryo-EM in complex with viral anti-CRISPR proteins [36] as well as a dsDNA target [38]. These structures revealed that the large subunit (Cas8f) of the type I-F Cascade features an additional N-terminal ‘hook’-domain. After sampling, the PAM sequence is sandwiched between the hook and the neighboring Cas proteins (i.e. Cas5f and Cas7.6f). Two additional structural motifs of the large subunit, a lysine-containing wedge and an alanine-rich loop specifically recognize the two base pairs of the PAM duplex. The lysine-wedge fulfills a similar purpose as the glutamine-wedge of Cas8e for strand separation but its tighter interactions with the first PAM base pair result in stricter PAM discrimination. The alanine-loop fulfills a similar role as the glycine-loop of Cas8e and recognizes the second base pair of the PAM element (Figure 2).

Cryo-EM structures of I-F Cascade bound to viral anti-CRISPR proteins have revealed that these small proteins can interfere with DNA binding. For example, AcrF2 and AcrF10 prevent access of the PAM duplex to the binding site of I-F Cascade. While AcrF2 partially overlaps with the dsDNA binding site, AcrF10 is considered to be a dsDNA mimic that directly blocks target recognition [36,38].

Recently, a minimal variant of the type I-F system (type I-Fv) has been discovered in *Shewanella putrefaciens* CN-32 and characterized [80,81]. Strikingly, small and large Cascade subunits are absent in this system and, consequently, all previously described elements for PAM recognition are missing. The system was shown to maintain activity and to rely on the presence of the same two G-C base pair PAM that is frequently observed for type I-F systems [80,81]. Type I-Fv Cascade consists of three Cas proteins. The 5′ repeat tag of the crRNA was shown to be bound by the protein Cas5fv and the crRNA spacer forms a complex with several copies of the backbone protein Cas7fv. Shortening the crRNA spacer resulted in the loss of several Cas7fv subunits and generated a more rigid Cascade assembly that was suitable for crystallization. The crystal structure of this complex was solved in absence and presence of an R-loop mimic [82]. The PAM motif was found to be read from the major groove side of the DNA, which is in contrast to previously observed minor groove side recognition of PAM elements by large Cascade subunits. The absence of a large subunit is compensated by the presence of an additional Cas5fv domain. This domain consists of six α-helices and reaches into the space of the large subunits of type I-F Cascade. The target DNA duplex is pinched between this alpha-helical domain and a small helix of Cas5fv. PAM read out is facilitated by an N-terminal linker and the α-helix 6 of Cas5fv. A set of amino acids directly interacts with the two base pairs of the PAM: a glutamine residue distorts the first G-C base pair and the second G-C base pair interacts with a lysine residue and an aspartate residue of the C-terminal helix of AH (Figure 2). Superimposition of I-Fv Cascade with and without target DNA revealed that a conformational shift pushes α-helix 3 of Cas5fv as a wedge against the first G-C base pair. Polar side chains of adjacent amino acids might assist in DNA strand separation. The non-target strand is then guided along a trench-route formed by Cas5fv and six Cas7fv subunits. These proteins possess two positively-charged loops that form a helix to guide the non-target strand in a fashion that resembles DNA guidance by small subunits of type I-E and I-F Cascade.

Much less is known about PAM recognition in other type I systems as structural data is missing. It is plausible that the universal presence of large subunits suggests that wedge insertion is commonly employed in different Cascade assemblies. Variations in PAM binding pockets allow for variable PAM sequences and different readout stringencies. The type I-A system (e.g. found in *Sulfolobus islandicus* and *Sulfolobus solfatarius*) has been shown to require a 5ʹ-CCN-3′ PAM motif for interference [83,84]. The highly promiscuous type I-B system was characterized for haloarchaeal model systems and shown to recognize six different PAMs: TTC, ACT, TAA, TAT, TAG, and CAC [85]. Type I-C systems are the second most abundant subtype and are characterized by a Cas5-dependent crRNA maturation pathway [86,87]. The PAM-SCANR method has been used to identify functional PAMs for this system in *Bacillus halodurans* and revealed an NTTC consensus PAM, matching previous bioinformatic analyses [88]. This consensus PAM represents the reverse complementary sequence of known PAM elements from type I-E, which suggests differences in the recognized DNA strands [51].

## PAM recognition by type II effector proteins

Type II CRISPR-Cas systems employ a single Cas9 effector protein, which also represents a highly popular genome engineering tool [6,89–91]. Cas9 proteins form a ribonucleoprotein complex with a crRNA and a second, trans-activating CRISPR RNA (tracrRNA). The artificial fusion of these molecules generates so-called single guide RNAs (sgRNA) that specify Cas9 cleavage sites [6,92,93]. Recognition of the target site requires complementarity to the crRNA spacer and the presence of a specific PAM sequence in the targeted region [16]. Customization of the guide RNA sequence allows for flexible targeting of different DNA regions and Cas9 has therefore been described as a programmable DNA scissor. However, the required interactions of Cas9 with PAM sequences limit the repertoire of possible targets and exclude certain genome engineering sites [94,95]. Consequently, in recent years many groups aimed to (i) elucidate PAM requirements for Cas9 variants of different organisms and (ii) engineer novel PAM specificities to broaden the targeting potential of Cas9.

*Streptococcus pyogenes* Cas9 (SpCas9) recognition of a short 5´-NGG-3´ PAM sequence has been studied in detail [16,53,96]. The crystal structure of SpCas9 revealed two major lobes, a recognition (REC) and a nuclease (NUC) lobe which accommodate the sgRNA:DNA hetero-duplex [97]. The NUC lobe does not only contain RuvC and HNH nuclease domains that are required for DNA cleavage, but also hosts the PAM interacting (PI) domain [97,98]. Binding of the tracrRNA-crRNA duplex results in extensive structural rearrangements of SpCas9, including the formation of the PAM interacting site [98–100]. This rearranged SpCas9 formation is competent for target recognition and interrogates DNA for the presence of correct PAM sequences. Two conserved arginine residues in the PAM-interacting (PI) domain have been shown to form major groove interactions with the non-target strand GG dinucleotide. Additional interactions with the minor groove of the PAM duplex help destabilizing the dsDNA target [19,97,98]. The recognition of the PAM initiates stable R-loop formation and allosterically regulates double stranded blunt DNA cleavage in a fixed distance of 3 bp upstream of the PAM [19,97,98,100–102]. Therefore, PAM recognition is a prerequisite for SpCas9 activity [19,101].

**Figure 3.**
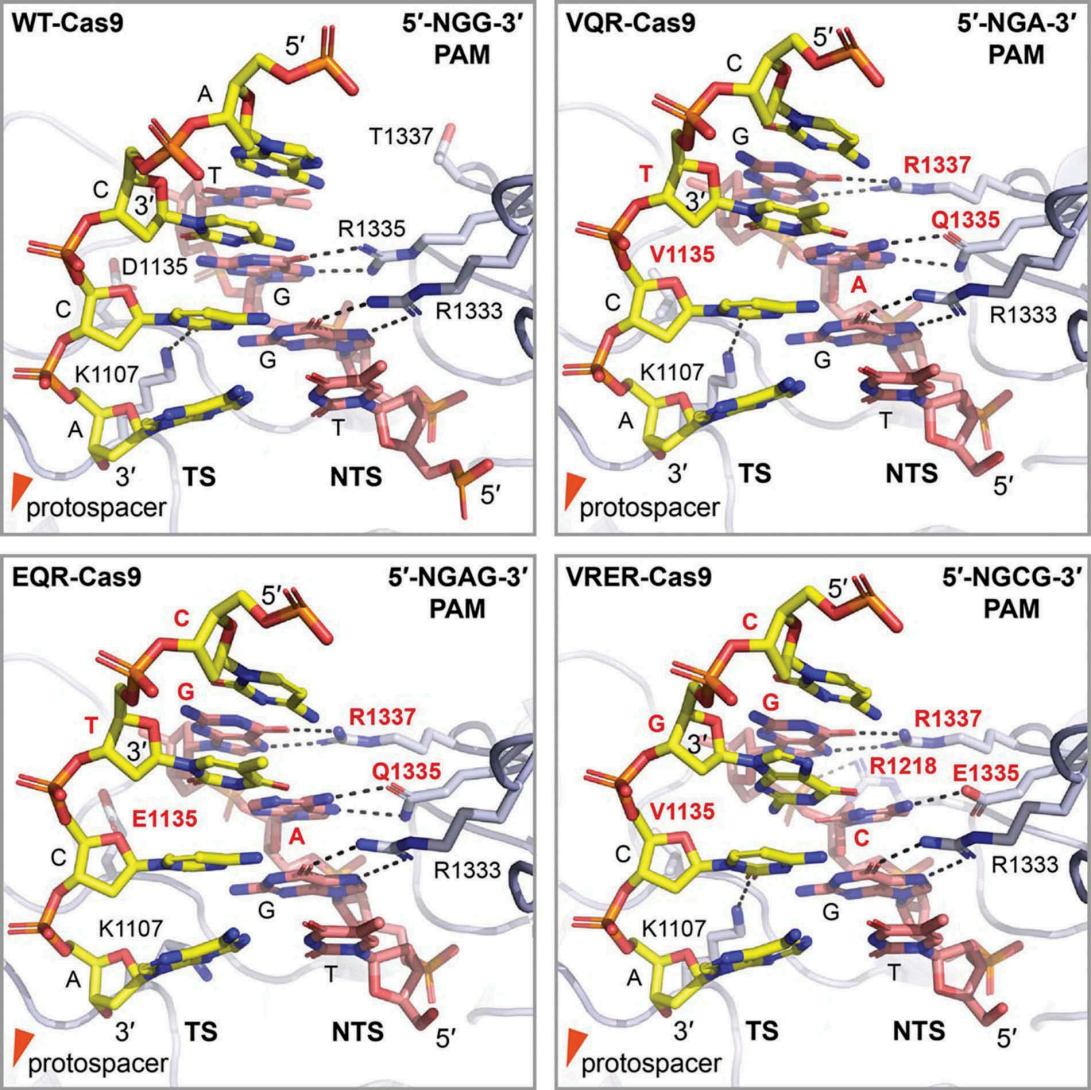
PAM recognition by wildtype and PAM specificity engineered Cas9 variants. Shown is the detailed view on the PAM interacting region of wildtype Cas9 (PDB: 4UN3 [98]) and the three engineered Cas9 versions VQR-Cas9, EQR-Cas9 and VRER-Cas9 (PDB: 5B2R, 5B2S and 5B2T [104]) in the respective panels. Cas9 (light blue) specifically recognizes the GG, NGA, NGAG and NGCG PAM respectively, mainly by polar interactions (black dashed lines) with the non-target strand (NTS, red colored) via the major groove of the PAM containing DNA duplex. K1107, with exception of the EQR-Cas9 variant, forms a hydrogen bond in the minor groove with the target strand (TS) cytosine of the first GC base pair of the PAM, further contributing to specificity. Multiple mutations in the engineered Cas9 variants (red label) result in displacement of the phosphodiester backbone of the PAM duplex and allow the side chain in position 1135 to recognize the altered third PAM nucleotide from the minor groove [104]. Altered PAM nucleotides are labeled red for clarity.

The requirement for specific PAM sequences limits the selection of possible target regions for genome-editing. To address this issue and to extend the active PAM range, PI domains were engineered. Substitution of the indicated two arginine residues with glutamine residues did not yield a proposed change of PAM specificity towards A-rich sequences [50,98]. Structure-guided directed evolution approaches were employed to mutagenize PI domains that were selected to interact with a NGA PAM. Three SpCas9 variants were obtained that harbored PI domains with altered PAM specificity: VQR (D1135V, R1335Q, T1337R), EQR (D1135E, R1335Q, T1337R) and VRER (D1135V, G1218R, R1335E, T1337R). The VQR variant exhibits a more flexible PAM recognition (NGAN & NGCG), while the EQR SpCas9 variant exhibits specificity for a NGAG PAM sequence. Finally, the VRER variant was found to be highly specific for NGCG PAM sequences. It has been calculated that these newly available PAM choices double the targeting potential of SpCas9 in human cells [50]. The crystal structures of these three SpCas9 variants revealed that they possess nearly identical conformations as the wildtype enzyme. The mutations result in the remodeling of the PAM region of the bound DNA, which is recognized by an induced fit mechanism [103]. Interestingly, in all three variants, substitution at position 1337 (T1337R) extends the recognized PAM sequence by one G nucleotide [103,104] (Figure 3).

This engineering approach has been extended to other orthologues of Cas9. The largest studied Cas9 ortholog was identified in *Francisella novicida* (FnCas9) and shown to recognize a canonical 5ʹ-NGG-3ʹ PAM sequence. Engineering of the PI domain produced a variant with a more flexible PAM of YG [105]. The smaller *Staphylococcus aureus* Cas9 ortholog was shown to recognize an extended PAM sequence (NNGRRT) and was engineered to a variant with more relaxed PAM recognition (NNNRRT) with high specificity for target cleavage in human cells described [106,107].

The analysis of the PAM specificity of naturally occurring Cas9 orthologues provides another means for broadening the target range of these DNA nucleases and the simultaneous use of different Cas9 enzymes with alternate PAM sequence requirements allows for multiplex genome editing applications. The DNA cleavage activity of eight representatives of phylogenetically defined type II CRISPR-Cas groups has been characterized and specific PAM sequences have been defined. Different tracrRNA-crRNA duplexes have been identified and were shown to be interchangeable between closely related type II systems of the PAM sequence was adjusted [108].

Anti-CRISPR proteins have also been identified for Cas9 proteins and were suggested to allow for modulation of Cas9-mediated genome editing approaches [109]. The anti-CRISPR protein AcrIIA4 was shown to mimic the PAM-containing DNA target and to occupy the PAM interacting domain and the RuvC domain of SpCas9 [37,39,40].

It was recently discovered that some Cas9 proteins also exhibit RNA-guided, PAM-independent RNase activities [110–112]. In addition to these natural RNase activities, canonical SpCas9 activity can also be redirected towards RNA substrates if the PAM is presented *in trans* on a separate DNA oligonucleotide. Short PAM-containing ssDNA molecules (termed PAMmer) are annealed to the target RNA and SpCas9-mediated target ssRNA cleavage is induced [113,114].

## PAM recognition by type v effector proteins

Type V CRISPR-Cas systems are defined by the stand-alone effector protein Cas12, containing a RuvC nuclease domain and a second putative nuclease domain instead of the HNH domain of the type II effector protein Cas9 [33,115–117]. Type V systems are subdivided into 5 groups, A to E and a predicted U subtype [118,119].

**Figure 4.**
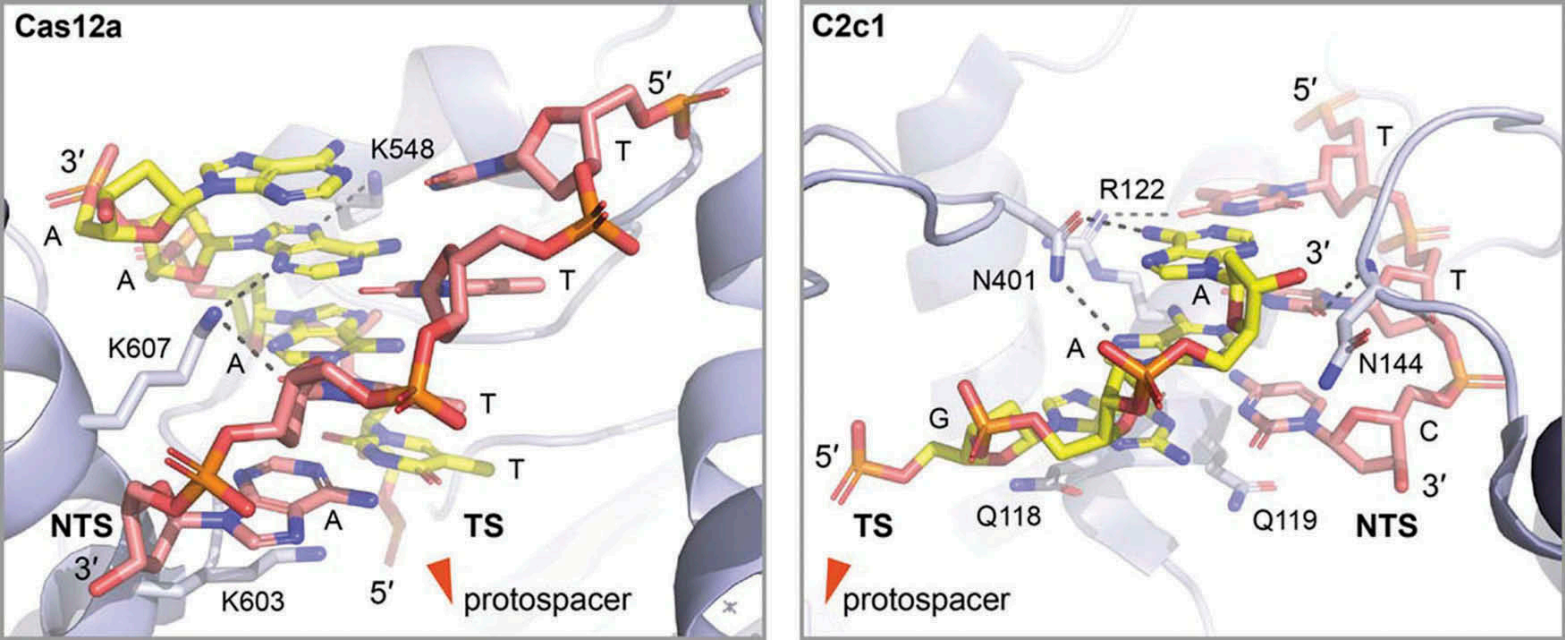
PAM recognition by type V Cas12a and C2c1. Left: Close up on the PAM interacting region of the *Acidaminococcus sp*. type V Cas12a (light blue) (PDB: 5B43 [117]). Cas12a tightly encloses and recognizes the TTTN PAM by a set of polar interactions from the minor and major groove of the PAM containing duplex. For clarity, van der Waals interaction mediating side chains are not shown. Notably, Lysine K603 stacks under the last nucleotide of the non-target strand (NTS, red), potentially assisting in target (TS, yellow) and non-target strand separation. Right: Close up on the PAM interacting region of the *Alicyclobacillus acidoterrestris* type V C2c1 (light blue) (PDB: 5U30 [116]). C2c1 tightly encloses and recognizes the TTC PAM by a set of polar interactions from the minor and major groove of the PAM containing duplex. For clarity, van der Waals interaction mediating side chains are not shown. Noteworthy, two glutamines (Q118 and Q119) stack under the last PAM base pair potentially assisting in target (TS, yellow) and non-target strand (NTS, red) separation.

Type V effector proteins share the same bilobal architecture of Cas9 proteins, but are usually smaller in size. Mechanistic differences to Cas9 activity are apparent as type V effectors recognize a T-rich PAM sequence and degrade their targets via staggered double strand breaks [13]. Target-bound structures have been elucidated for the interference modules Cas12a (Cpf1) and Cas12b (C2c1), providing insights into their PAM recognition mechanism [115–117,120,121]. Cas12a is guided by a single crRNA, without the need for a tracrRNA [13]. This stand-alone protein is able to process its precursor RNA and target DNA with a canonical 5′-TTTN-3′ PAM [122]. As described for *Acidaminococcus sp*., AsCas12a PAM recognition depends on major groove interactions with the wedge (WED) and recognition 1 (REC1) domains, plus minor groove interactions with the PAM-interacting (PI) domain. For the PAM −1 position, the base pair does not form base-specific contacts with the protein, allowing a promiscuous read at this position. At position −2, hydrogen bonds between the non-target strand dT and Lys607, plus Van der Waals forces between the target strand dA and the recognition pocket ensure T-A pair recognition. Hydrogen bonds also form upon PAM insertion in the positively charged central channel of the protein, between the non-target dA (−4) with its pair dT (−4) and dA (−3) (Figure 4). Taken together, these interactions account for both base and shape readout of the PAM, which is in contrast to base-only readout by Cas9. As residues involved in these interactions are conserved in the Cas12a family, it is suggested that PAM recognition occurs in a similar manner in different variants [117,123].

In order to evaluate the use of AsCas12a and *Lachnospiraceae bacterium* Cas12a (LbCas12a) for genome editing, a high-throughput system was developed that allowed testing crRNA libraries in mammalian cells [124]. Here, target specificity of different RNA/target pairs correlated with the frequency of observed insertions and deletions (indel) at the target site. Surprisingly, the fourth position of the PAM was found to exclude Ts, redefining the canonical PAM for this system as TTTV. In addition, CTTA-PAM showed the highest indel frequency besides TTTV, highlighting this sequence as a secondary PAM option. Furthermore, LbCas12a showed higher indel frequency when a C nucleotide flanked the canonical TTTA PAM (CTTTA) [124].

In the light of these results, Yamano and collaborators set out to determine the mechanisms behind non-canonical PAM recognition [120]. For this, the crystal structures of LbCas12a bound to target sequences flanked by TTTA, TCTA, TCCA, or CCCA PAMs were determined. Overall, the domain composition of LbCas12a does not differ from AsCas12a. When compared to the AsCas12a binary complex, DNA binding elicits a conformational change of the protein as the PI domain moves towards the REC1 and WED domains to form the PAM-binding channel. For canonical PAM recognition, LbCas12a differs from AsCas12a by a more stringent read out of PAM (−2). The base and shape PAM recognition mechanism is conserved between these two proteins. For the degenerated PAMs, the crystal structures show that the sub-optimal readout is made possible by the flexibility of the PI domain, while REC1 and WED domains remain unchanged. The PI domain undergoes an outward displacement, resulting in an opening of the PAM binding cannel [120].

Cas12a is frequently employed for genome editing approaches with benefits including its small size, tracrRNA independency and asymmetric cleavage sites. In order to expand the range of possible Cas12a targets, several protein variants with different PAM specificities were designed [125]. The targeting activity of AsCas12a with single residue mutations in the PAM pocket was assessed via plasmid depletion assays in *E. coli* and indel frequency assays in mammalian cells. These approaches yielded mutants S542R/K607R (RR) and S542VR/K548V/N552R (RVR) which showed the most suitable activities. The AsCas12aRR mutant cleaves specifically at TYCV PAM sites, while the RVR mutant recognizes TATV PAM targets. Both variants were found to exhibit higher activity than the wild type AsCas12a. These new motifs expand the targeting range of the protein to one target site for every 11 bp stretch in human coding sequences. The introduction of the additional mutation K949A reduced off-target effects. Conservation of the mutated residues among members of the Cas12a family suggest a general approach for broadening the range of genome editing targets [125].

Recently, two new type V effectors with a single conserved RuvC domain were identified and termed C2c1 (Cas12b, found in 83 genomes) and C2c3 (Cas12c), identified from metagenomic data) [118]. For C2c1, dsDNA-targeting activity relies on a tracrRNA and a crRNA. A T-rich PAM sequence was identified for this system. For the *Alicyclobacillus acidoterrestris* C2c1 (AacC2c1) enzyme, recognition of TTT, TTA and TTC PAM sequences was proven *in vivo* [118]. The PAM specificity is consistent with the TTN PAM of Cas12a. The crystal structure of AacC2c1 shows a bilobal composition that resembles the architecture of Cas12a, with both a conserved RuvC nuclease and a divergent NUC domain. A PI domain is absent. PAM recognition was shown to occur between two domains (termed OBD and Helical-1) in the two lobes, with motif readout through shape and base interactions at the major and minor DNA grooves. The first two T residues of the PAM are read through base-specific contacts (forming hydrogen bonds with several residues), while the third promiscuous position is stabilized through base-independent stacking between two glutamines, that also help R-loop formation (Figure 4). In contrast to PI domain closure upon target binding in Cas12a, C2c1 has a pre-organized cleft that undergoes a disordered to ordered change in order to read out the PAM in a ‘locked’ state [116]. This recognition mechanism is considered to be more stringent and could avoid off-target effects [126].

Little is known about the target specificity of other type V effectors. The type V-C protein C2c3 (Cas12c) is characterized by the absence of a tracrRNA plus particularly short spacers, which complicates the search for protospacers and associated PAMs [119]. Small variants of type V CRISPR-Cas systems were identified in metagenomic datasets, and their signature proteins were denominated CasY (Cas12d) and CasX (Cas12e). CasY has C2c3 as its closest relative, acts tracrRNA-independent and is encoded next to a CRISPR array characterized by small spacers (17–19 bp). A TA PAM requirement was identified via plasmid depletion assays. For CasX, a 5ʹ-TTCN-3ʹ PAM was described and a tracrRNA is needed for interference [127]. Taken together, type V proteins and their PAM recognition mechanisms reveal a structural diversity while maintaining functional convergence [33]. Many type V effector complexes exhibit variable and uncharacterized domains. Further structural studies will help to elucidate the full range of PAM recognition mechanisms in these proteins.

## Prevention of autoimmunity in type III crispr-cas systems

Type III CRISPR-Cas systems are widely distributed in Archaea and also found in some Bacteria. They are divided into 4 subtypes (A-D) and are usually found in conjunction with type I systems. Type III-A systems usually carry an adaptation module, whereas most of III-B systems do not and depend on others systems to incorporate new spacers. Furthermore, the adaptation genes are also not found in type III-C and D loci [86]. Similar to type I systems, type III interference is carried out by crRNA-guided multi-protein complexes, termed Csm for subtypes A and D, and Cmr for subtypes B and C [86].

Mechanistically, interference in type III diverged from type I systems by their ability to degrade DNA in a transcription-dependent manner [128–131]. Most Csm1 and Cmr2 proteins (Cas10 family) have a HD nuclease domain and a GGDD motif responsible for ssDNA degradation [132–134], while Csm3 and Cmr4 (Cas7 family) exhibit endoribonuclease activity for sequence-specific RNA degradation [134–137]. Cas10 is allosterically activated upon crRNA-RNA binding resulting in the co-transcriptional cleavage of target DNA and its transcripts [138]. An initial study described a degenerated RNA PAM dependency for the Cmr complex of *Pyrococcus furiosus* [128]. Other reports on type III-A and B activities support that self-targeting by Cas10 is prevented as long as the 5′-end of the crRNA is complementary to the 3′-flank of the target RNA protospacer [129,130,139]. In these systems, the hybridization of only three or two bases of the 8 nt 5′-handle of the crRNA is sufficient to block target degradation [25,130]. For type III-Bv, at least 4 bases of the unusually long 13- or 14- nucleotide long handle must be unpaired for the system to be active [140]. In these systems, this method for non-self-discrimination is sufficient, since PAM elements are not required to specify the sites dsDNA opening. Although the low stringency of these interactions might facilitate the escape of viral targets, it could have been selected in order to keep the unspecific DNase activity of the complex under control. Also, as type III systems are usually found together with type I systems, the PAM-independent recognition broadens the targeting spectrum and might help to catch PAM escape mutants that evade Cascade interference [141].

## Target recognition by type VI rnases

The type VI CRISPR-Cas system contains single-effector RNA-guided RNases that have been classified into 4 subtypes. All known systems act without tracrRNAs and the effectors possess little sequence homology apart from two HEPN (Higher Eukaryotes and Prokaryotes Nucleotide-binding) domains, which are typically found in other proteins with RNase activity.

Cas13a (C2c2) was the first protein from this type to be characterized. Its HEPN domains adopt a unique fold which is conserved among other type VI proteins [14,118]. It is able to perform pre-crRNA maturation with a previously uncharacterized third nuclease domain [142]. The RNA-targeting activity was first shown for *Leptotrichia shahii* Cas13a (LshCas13a), which was able to provide protection against the ssRNA virus MS2 when heterologously expressed in *E. coli* [14,143]. This activity relied on the presence of a Protospacer Flanking Site (PFS), which represents an analogue to PAMs for RNA targets. Specific discrimination against G at the 3′-end of target RNA was observed. The presence of a C at the corresponding crRNA repeat site indicates that nucleotide pairing at this position is rejected. After target binding, this protein is able to carry out target and collateral ssRNA cleavage at uracil sites. This is proposed to trigger programmed cell death, as LshCas13a activity generates growth defects in *E. coli* [14,143]. The trans-acting RNA cleavage was recently repurposed for RNA detection, as the indiscriminate degradation acts as signal amplification [144].

LshCas13a apo and target-bound structures revealed a bilobal protein with REC and NUC domains without homology to known Cas protein domains [145]. The *Leptotrichia buccalis* Cas13a (LbuCas13a) RNA-bound structure reveals that the PFS is discriminated through the formation of a hydrogen bond with Lys47, avoiding base pairing at position −1 [146].

PFS sequence preferences were not observed for *Leptotrichia wadei* Cas13a (LwaCas13a) and *Prevotella sp*. P5-125 (PspCas13b) proteins in mammalian cell interference, which correlates with higher activity against target RNAs than LshCas13a [143,147].

Type VI-B proteins are predicted to have evolved from trans-membrane proteins, as they contain corresponding trans-membrane domains that set them apart from other type VI proteins [89,148]. For *Bergeyella zoohelcum* Cas13b (BzCas13b), PFS identification at both target sites was recently described, with 5′-recognition of D (G, T, A) and a 3′-motif requirement of NAN or NNA. In addition, RNA accessibility was shown to play a relevant role in target recognition [148]. Overall, type VI systems seem to follow less restrictive rules for substrate recognition than other types, as its sole RNA targeting activity is expected to have less detrimental effects on the cell upon self-targeting.

## Conclusions

Most activities of Cas protein DNases and RNases require short sequence motifs (PAM or PFS) to identify proper targets and prevent self-cleavage of the host genome. Relaxed target selection mechanisms rely on the absence of complementarity between crRNA tags and the protospacer. However, most CRISPR-Cas systems identify specific PAM sequences and a large variety of PAM-interacting domains has been described for Cas protein effector complexes. Modulation of the sequence or accessibility of PAM elements renders these CRISPR-Cas systems ineffective. Consequently, viruses have been observed to mutate or modify PAM sequences and to evolve anti-CRISPR proteins that target PAM-interacting regions. In a possible response, CRISPR-Cas systems evolved a large variety of PAM-readout strategies to enable targeting of virtually any viral sequence. Recent structural studies highlight the diversity of mechanisms and domain architectures that are employed to guarantee target specificity. Future work will reveal novel PAM-interaction modules by yet uncharacterized Cas proteins. The applicability of these enzymes for genome engineering approaches relies on the availability of PAM sequences in the target region. Consequently, protein-engineering approaches will be extended to create designer PAM-interaction domains with a desired range of selectable targets.
